# Effects of oral contraceptives on selected parameters of the homeostatic control system in young women having a sudden disorder of the auditory and/or balance system

**DOI:** 10.1007/s00405-013-2853-x

**Published:** 2013-12-12

**Authors:** Joanna Urbaniak, Hanna Zielińska-Bliźniewska, Jarosław Miłoński, Piotr Pietkiewicz, Krzysztof Kuśmierczyk, Jurek Olszewski

**Affiliations:** Department of Otolaryngology and Laryngological Oncology, Medical University of Lodz, Żeromskiego 113, 90-549 Lodz, Poland

**Keywords:** Oral contraceptives, Parameters of the homeostatic control system, Auditory and balance disorders

## Abstract

The purpose of the paper was to assess the effects of oral contraceptives on selected parameters of the homeostatic control system in women having a sudden disorder of the auditory and/or balance system. The study included 105 young women divided into two groups: Group I—52 women with the disorder of the auditory and/or balance system using hormonal contraceptives for at least 2 months, aged 20–49; and Group II—53 women without any disorder of the auditory and/or balance system using hormonal contraceptives for at least 2 months, aged 18–40. The patients included in the study underwent a full otoneurological evaluation, detailed laryngological diagnostics and an evaluation of selected parameters of the homeostatic control system—fibrinogen level, D-dimer level, evaluation of APTT and PT indicator, plasma estradiol and progesterone with the Roche Cobas analyser by means of chemiluminescence. The vertigo occurring in the study group was most often central (59.6 % of cases), mixed with compensation in 36.6 % of cases, and peripheral only in 3.8 % of cases, indicating labyrinth damage in 40.4 % of cases. An analysis of the progesterone level, considering the menstrual cycle phase in the group, showed that its value was abnormal in 51.0 % of women in the study group and 47.1 % in the control group. In their own studies, the authors observed that the estradiol level in the plasma, considering the menstrual cycle phase in the study group, was abnormal in 41.2 % of women and that the differences in its concentration were statistically significant in the study and control groups (*p* = 0.005), which may have a negative impact on the possibility of a thromboembolic episode.

## Introduction

Since the 1960s oral contraceptives have been a well-known risk factor for thromboembolic episodes. Studies indicate that in women taking contraceptives the risk of venous thrombosis increases by 3–6 times. The convenience and reliability of this contraceptive method are the reasons why the pharmaceutical industry continues to improve it and performs research on new agents.

The most common reason for the discontinuation of contraception by women is the occurrence or fear of adverse effects. The most severe ones are thromboembolic complications that may be a direct cause of death. The most severe conditions that are directly life or health-threatening to the patient include: myocardial infarction, pulmonary embolism and cerebrovascular accident.

A theory was put forward to explain oestrogen/progesterone complex conditioning the risk of thrombus. The joint action of these two hormones was said to have an ultimate effect on coagulation and fibrinolysis. A decrease in the estradiol dose reduced the risk of thromboembolic complications, while the contraceptive effect was maintained. It was also confirmed that third-generation drugs were considered to have a greater, total “oestrogen” effect, and thus thrombus-promoting effect, than second-generation drugs [1–6].

A thromboembolic episode is observed most frequently during the first year, or even first 3 months, of taking hormonal contraceptives. The most probable cause seems to be the concurrence of a pre-existing genetic or environmental factor with the additional factor of hormonal contraception, leading to the pathological activation of the coagulation system.

Currently, factors increasing the risk of thromboembolic episodes by 2–5 times are considered strong. The genetic factors of coagulation disorders include a deficit of naturally occurring anticoagulant proteins, such as antithrombin, protein C, protein S, and mutation of factor V Leiden related to the resistance to the anticoagulative effect of protein C. Additionally, it needs to be emphasised that weak, accumulating hereditary risk factors of thromboembolic disease increase the total risk of a disorder in the coagulation system. They include having a blood type other than O, certain genetic variants of prothrombin and fibrinogen, and factor XIII or XI [7–9].

There are also mutations of proteins in the homeostatic system, with anticoagulative effects and which decrease the pro-coagulation properties.

Hormonal contraceptives increase the level of the following coagulation factors (Tables 1, 2): IX, X, XI, prothrombin, and fibrinogen, while decreasing the level of anticoagulative factors, e.g. antithrombin, proteins C and S, thus disturbing the delicate balance and potentially leading to thromboembolic disease [10, 12].

**Table 1:** Biochemical and laboratory results in the study group

	Statistical parameter						
	M	SD	SE	95 % CI	Quartiles	Min.	Max.
Estradiol (pg/ml)	312.29	288.90	40.06	231.86–392.72	124.46;222.18;377.91	23.35	1,306.40
Follicular p.	404.09	306.26	74.64	248.88–559.31	145.79;236.77; 629.54	37.92	1,306.40
Ovulation peak	210.75	92.63	29.98	133.69–287.82	154.70;185.93; 276.60	139.15	322.22
Luteal p.	243.53	259.24	50.11	139.60–347.46	73.26; 226.60; 348.87	23.35	1,077.00
Progesterone (ng/ml)	3.28	3.51	0.49	2.30–4.25	0.80; 1.69; 5.00	0.29	15.00
Follicular p.	2.13	1.94	0.55	0.97–3.28	0.69; 1.13; 2.06	0.56	12.10
Ovulation peak	5.52	4.13	1.83	0.81–10.23	1.45; 5.28; 8.62	0.79	11.70
Luteal p.	3.88	3.12	0.78	2.26–5.51	0.79; 2.87; 6.60	0.29	15.00
D-dimers (μg/ml)	0.69	2.42	0.34	0.02–1.37	0.20; 0.31; 0.44	0.19	17.76
Fibrinogen (mg/dl)	245.12	67.03	9.30	226.45–263.78	208; 239.50; 278	89	395
APTT (s)	30.81	3.03	0.42	29.97–31.65	28.60; 30.55; 32.35	25.80	39.20
PT (s)	12.00	0.90	0.13	11.75–12.25	11.40; 11.80; 12.55	10.10	14.30

**Table 2:** Biochemical and laboratory results in the control group

	Statistical parameter						
	M	SD	SE	95 % CI	Quartiles	Min.	Max.
Estradiol (pg/ml)	214.81	256.76	35.27	144.04–285.58	64.03;106.68;218.20	23.35	1,012.90
Follicular p.	245.00	251.70	47.57	147.40–342.60	73.69;138.03;363.23	33.97	948.23
Ovulation peak	67.05	15.28	7.64	42.74–91.37	58.00; 72.84; 76.11	44.49	78.04
Luteal p.	202.70	282.92	61.74	73.92–331.48	39.04; 74.53; 203.98	23.35	1,012.90
Progesterone (ng/ml)	1.34	1.27	0.17	0.99–1.69	0.67; 0.87; 1.49	0.13	6.66
Follicular p.	1.24	1.10	0.21	0.81–1.67	0.75; 0.82; 1.30	0.34	6.05
Ovulation peak	0.86	0.33	0.16	0.34–1.38	0.61; 0.80; 1.11	0.55	1.29
Luteal p.	1.57	1.56	0.34	0.86–2.28	0.51; 1.27; 1.75	0.13	6.66
D-dimers (μg/ml)	0.36	0.45	0.06	0.23–0.48	0.19; 0.21; 0.34	0.19	3.39
Fibrinogen (mg/dl)	238.68	54.48	7.48	223.66–253.69	195; 231; 261	151	387
APTT (s)	29.78	2.10	0.29	29.20–30.35	28.40; 29.50; 31.30	24.60	34.00
PT (s)	12.06	0.63	0.09	11.88–12.23	11.60; 12.10; 12.60	10.80	13.40

The purpose of the paper was to assess the effects of oral contraceptives on selected parameters of the homeostatic control system in young women with a sudden disorder of the auditory and/or balance system.

## Materials and methods

The studies included 105 women hospitalised in the Department of Otolaryngology and Laryngological Oncology of the Military Medical Academy University Teaching Hospital in Lodz, divided into two groups:

I—52 women with a disorder of the auditory and/or balance system, using hormonal contraceptives for at least 2 months, aged 20–49 (mean age 33.31 ± 5.85 years),

II—53 women without the disorder of the auditory and/or balance system, using hormonal contraceptives for at least 2 months, aged 18–40 (mean age 29.13 ± 5.78 years).

The patients in the study underwent the following procedures:

- Detailed otoneurological and gynaecological history, especially with regard to: use of hormonal contraceptives, duration of treatment, type of agent used, use of anticoagulants, and concurrent liver diseases. The studies did not include patients taking anticoagulants or those with a history of thromboembolic disease. Any possible liver diseases were excluded by laboratory tests.
- Physical laryngological and otoneurological examination.
- Blood test (blood count, CRP, electrolytes, glucose level and lipid profile) to exclude metabolic diseases; hepatic enzyme assessment (ALAT, ASPAT) to exclude liver diseases as a potential cause of homeostatic disorders.
- Otoneurological diagnostics were performed, including videonystagmography test (VNG), which included recording of the spontaneous nystagmus, pendular nystagmus, positional nystagmus, optokinetic nystagmus, kinetically induced nystagmus using the swivel chair test and a neck torsion test.
- Audiometric examination, including: determination of the noise level, tone threshold and suprathreshold audiometry, speech audiometry, and brainstem evoked response audiometry (BERA). In selected cases acoustic otoemission (TEOAE and DPOAE) and impedance audiometry were performed: tympanometry and recording of the stapedius reflex, with determination of the reflex threshold at the frequencies of 500, 1,000, 2,000 and 4,000 Hz, with stimulation of the ipsi- and contralateral pure tone and the Reflex Decay Test.
- Computer tomography or magnetic resonance of the head to exclude any organic lesions.
- Doppler blood flow test in spinal arteries and the basilar artery was performed with Tranpect-TCD, 2 MHz head (entrance window—foramen magnum) and the neck torsion test (to exclude vascular causes).
- Tests of selected parameters of the homeostatic system (assessment of the fibrinogen level, D-dimer level, assessment of the APTT and PT indicator), performed by the Department of Laboratory Diagnostics and Clinical Biochemistry of the Military Medical Academy University Teaching Hospital in Lodz, based on the Sysmex CA-560 System by SIEMENS.
- Tests of the plasma estradiol and progesterone levels performed at the Endocrinology Clinic of the Endocrinology Department of the Medical University of Lodz, with the Roche Cobas analyser by means of chemiluminescence. The results for reproductive hormones included differences in concentration depending on the menstrual cycle.

Those women with a sudden auditory disorder were treated with oral glucocorticoids, with the protective administration of proton pump inhibitors, vascular drugs and drugs improving the metabolism of the cerebral tissue (in the acute phase by intravenous drip, and orally after 5–7 days) for approx. 30 days.

On the other hand, those women with sudden vertigo were treated with antiemetic drugs only at the acute phase; vascular drugs and drugs improving the metabolism of the cerebral tissue, and blockers of the H_3_ autoreceptor.

The obtained results were subjected to statistical analysis using the following procedures (significance tests):

- Chen–Shapiro test (to estimate the normality of the measurable variable distribution),
- Levene test (to estimate the equality of variances),
- Mann–Whitney–Wilcoxon rank sum test,
- *χ* ^2^ independence test,
- Fisher exact test,
- logistic regression.

The test results were considered statistically significant if the significance level was *p* < 0.05.

## Results

In the study group (I), vertigo was: central in 31 (59.6 %) patients (*f* = 0.5769); peripheral with compensation in 1 (1.9 %) patient (*f* = 0.0385); peripheral without compensation in 1 (1.9 %) patient (*f* = 0.0192) and mixed with compensation in 19 (36.6 %) patients (*f* = 0.3654). Thus, labyrinth damage was diagnosed in 21 (40.4 %) patients in the study group, including the right labyrinth in 12 patients (23.1 %) and the left one in 9 patients (17.3 %).

On the other hand, auditory damage according to CPT was 0.4–97.0 % (mean value 5.87 ± 16.69 %) for the right ear, and from 0.1 to 53.0 % (mean value 3.47 ± 8.14 %) for the left ear.

The tested estradiol level in the blood serum (pg/ml), considering the menstrual cycle in the study group (I) was as follows: below normal in 6 (11.8 %) patients (*f* = 0.1176), normal in 30 (58.8 %) patients (*f* = 0.5882) and above normal in 15 (29.4 %) patients (*f* = 0.2941), whereas in the control group (II), in 19 (35.8 %) patients (*f* = 0.3585), in 28 (52.8 %) patients (*f* = 0.5283) and in 6 (11.3 %) patients (*f* = 0.1132), respectively. A statistical dependence of the estradiol level in the blood serum between the study and control group was found (*p* = 0.005).

The tested progesterone level in the blood serum (pg/ml), considering the menstrual cycle in the study group (I) was as follows: below normal in 12 (23.5 %) patients (*f* = 0.2353), normal in 25 (49.0 %) patients (*f* = 0.4902) and above normal in 14 (27.5 %) patients (*f* = 0.2745), whereas in the control group (II), in 19 (35.8 %) patients (*f* = 0.3585), in 28 (52.9 %) patients (*f* = 0.4717) and in 6 (11.3 %) patients (*f* = 0.1698), respectively. A statistical dependence of the progesterone level in the blood serum between the study and control group was not found.

The tested D-dimer level in the blood serum (μg/ml) in the study group (I) was as follows: normal in 42 (80.8 %) patients (*f* = 0.8077) and above normal in 10 (19.2 %) patients (*f* = 0.1923), whereas in the control group (II) in 43 (81.1 %) patients (*f* = 0.8113) and above normal in 10 (18.9 %) patients (*f* = 0.1887), respectively. A statistical dependence between the D-dimer level in the blood serum in the study and control group was not found.

The fibrinogen level in the blood serum (mg/dl) in the study group (I) was as follows: below normal in 10 (19.2 %) patients (*f* = 0.1923) and normal in 42 (80.8 %) patients (*f* = 0.8077), whereas in the control group (II) in 14 (26.4 %) patients (*f* = 0.2642) and normal in 39 (73.6 %) patients (*f* = 0.7358), respectively. A statistical dependence of the fibrinogen level in the blood serum between the study and control group was not found.

The prothrombin time (s) tested in the study group (I) was as follows: below normal in 45 (86.5 %) patients (*f* = 0.8654) and normal in 7 (13.5 %) patients (*f* = 0.1346), whereas in the control group (II) in 50 (94.3 %) patients (*f* = 0.9434) and in 3 (5.7 %) patients (*f* = 0.0566), respectively. A statistical dependence of the prothrombin time in the blood serum between the study and control group was not found. The tested APTT coagulation time was normal in all women in the study and control groups.

In the study group (I), the biochemical and laboratory results were as follows (Table 1): mean serum estradiol—312.29 ± 92.63 pg/ml, in the follicular phase—404.09 ± 306.26 pg/ml, in the ovulation peak—210.75 ± 288.90 pg/ml and in the luteal phase—243.53 ± 259.24 pg/ml; mean blood progesterone—3.28 ± 3.51, 2.13 ± 1.94, 5.52 ± 4.13 and 3.88 ± 3.12 pg/ml, respectively; D-dimer serum level was 0.69 ± 2.42 μg/ml; the fibrinogen level was 245.12 ± 67.03 mg/dl, respectively; APTT—30.81 ± 3.03 s and PT—12.00 ± 0.90 s.

On the other hand, in the control group (II) the biochemical and laboratory results were as follows (Table 2): mean serum estradiol—214.81 ± 256.76 pg/ml, in the follicular phase—245.00 ± 251.70 pg/ml, in the ovulation peak—67.05 ± 15.28 pg/ml and in the luteal phase—202.70 ± 282.92 pg/ml; mean blood progesterone—1.34 ± 1.27, 1.24 ± 1.10, 0.86 ± 0.33 and 1.57 ± 1.56 pg/ml, respectively; D-dimer serum level was 0.36 ± 0.45 μg/ml; the fibrinogen level was 238.68 ± 54.48 mg/dl, respectively; APTT—29.78 ± 2.10 s and PT—12.06 ± 0.63 s.

A statistical dependence was observed in the differences in estradiol level in the follicular phase and ovulation peak (*p* = 0.020 and *p* = 0.010) as well as for progesterone in the ovulation peak and the luteal phase (*p* = 0.038 and *p* = 0.032) between the study and control groups.

## Discussion

The causes of vertigo are relatively well-researched thanks to highly specialised diagnostic tests. However, for a large group of patients the tests performed so far have not shown any abnormalities, thus making it impossible to indicate a direct cause for the vertigo [13].

In the literature, the most common background is vascular (50–70 %), viral (12–25 %) and autoimmunological (18 %).

The combined contraceptive pill has been available in pharmacies for more than 50 years. Owing to its high effectiveness (Pearl index approx. 0.5 %), full reversibility of its effects and high acceptability by women, it has become the most universal contraceptive method [14]. Currently, it is estimated that approx. 100 million women take the combined contraceptive pill.

Giacomini et al. [15] believe that in patients taking oral contraceptives, the direct cause of suffering from mild paroxysmal vertigo is a disturbed electrolyte balance, changes in endolymph pH, and disturbed metabolism of glucose and lipids resulting from the use of hormonal contraceptives, confirmed by the authors’ own studies.

According to Mitre et al. [16] the use of hormonal contraceptives may contribute to the occurrence of tinnitus or disorders of the vestibular system. No negative impact on hearing has been observed.

A slightly moderate hearing loss according to the CPT of 5.87 ± 16.69 % for the right year and of 3.47 ± 8.14 % for the left year was observed in the authors’ own studies.

In the studies by Mueck et al. [17], the negative impact of oral contraceptives on diseases of the inner ear was not confirmed. Similar conclusions are drawn from the studies of Vessey et al. [6], who also believe that drugs containing less than 50 μg of the oestrogen component have a lower impact on thromboembolic episodes.

Lu et al. [18] concluded that in patients with sudden deafness, the levels of total cholesterol, triglycerides and lipoproteins were significantly higher compared to the control group. This may be an additional factor influencing blood viscosity, decreasing the blood supply to the cochlea.

In another report, Lu et al. [19] proved that in patients with sudden deafness, the level of fibrinogen and viscosity of the blood plasma were significantly higher compared to the control group. This may promote thrombophilia and decrease the blood supply to the cochlea.

The serum level of estradiol and progesterone depends on the menstrual cycle. In the study group (I) the patients were: in the follicular phase in 43.1 % of cases, in the ovulation peak in 11.8 % of cases, and in the luteal phase in 45.1 % of cases, whereas in the control group (II) in 52.8, 7.5 and 39.6 % of cases, respectively.

The authors’ own studies found that the estradiol level in the blood serum, considering the menstrual cycle phase in the study group (I) was as follows: below normal in 11.8 % of the patients, normal in 58.8 % of the patients and above normal in 29.4 % of the patients, whereas in the control group (II) in 35.8 % of the patients, in 52.8 % of the patients and in 11.3 % of the patients, respectively.

An analysis of the progesterone level in the blood serum, considering the menstrual cycle phase in the study group (I), gave the following values: below normal in 23.5 % of the patients, normal in 49.0 % of the patients and above normal in 27.5 % of the patients, whereas in the control group (II) in 35.8 % of the patients, in 52.9 % of the patients and in 11.3 % of the patients, respectively.

The studies by Vliet et al. [20] show that a greater oestrogen level in hormonal contraceptives contributes to an increased level of sex hormone-binding proteins (SHBPs). The impact of contraceptives on the increased level of SHBPs may be a risk indicator for a thromboembolic event.

Sitruk-Ware [21] states that there are great differences between individual progesterone components used in contraceptives, and that the final effect of a progesterone component depends on the type and dose of the combined oestrogen.

It has been shown that oral contraceptives influence the level of almost every protein of the blood coagulation system [10, 22].

Considering the delicate balance between the coexisting components of the coagulation and fibrinolytic system, even the slightest variation in the level of proteins participating in the coagulation process may launch a cascade. In the performed studies, it has been proven numerous times that oral contraceptives, depending on the generation and progesterone component, increase the level of fibrinogen, prothrombin, and coagulation factors VII, VIII and X, while decreasing the level of factor V, antithrombin, and the concentration and activity of the tissue factor pathway inhibitor (TFPI) [11, 23, 24]. Moreover, it has been proven that taking third-generation oral contraceptives is associated with a greater risk of thromboembolic disease than using older, second-generation drugs.

In the authors’ own studies, the D-dimer level in the blood serum in the study group (I) was as follows: normal in 80.8 % of the patients and above normal in 19.2 % of the patients, whereas in the control group (II) in 81.1 % of the patients and in 18.9 % of the patients, respectively.

The evaluated fibrinogen level in the blood serum in the study group (I) was as follows: below normal in 19.2 % of the patients and normal in 80.8 % of the patients, whereas in the control group (II) in 26.4 % of the patients and in 73.6 % of the patients, respectively. The prothrombin time (s) tested in the study group (I) was as follows: below normal in 86.5 % of the patients and normal in 13.5 % of the patients, whereas in the control group (II) in 94.3 % of the patients and in 5.7 % of the patients, respectively. The tested APTT coagulation time was normal in all women in the study and control groups.

As shown in the available publications, oral contraceptives increase the risk of thromboembolic episodes manifesting as an ischemic stroke or myocardial infarction, while the main factor responsible for the formation of thrombi is the oestrogen component level in the pill. Recent publications have been describing an oestrogen/progesterone complex increasing the risk of thrombi and embolisms [11, 24]. In their own studies the authors observed an abnormal estradiol level in the serum of 51 % of the patients, which may be a reason for the activation of the coagulation cascade.

Tanis et al. [25] emphasise the increased risk of thromboembolic disease during the use of contraceptives in patients who smoke, have hypertension, diabetes or hypercholesterolemia.

In his report, Stenchever [26] states that the prevalence of thromboembolic disease is related to the dose of oestrogen.

Contraceptives that have a smaller dose of oestrogens (20 μg) pose a lower risk of thromboembolic disease, as indicated by Lidegaard et al. [27].

## Conclusions

1. The vertigo occurring in the study group was most often central (59.6 % of cases), mixed with compensation in 36.6 % of cases, and peripheral only in 3.8 % of cases, indicating labyrinth damage in 40.4 % of cases.
2. In their own studies, the authors observed that the plasma estradiol, considering the menstrual cycle phase in the study group, was abnormal in 41.2 % of the patients and that differences in its concentration were statistically significant in the study and control groups (*p* = 0.005), which may have a negative impact on the possibility of a thromboembolic episode.
3. An analysis of the progesterone level, considering the menstrual cycle phase in the group, showed that its value was abnormal in 51.0 % of patients in the study group and 47.1 % in the control group.
4. A statistical dependence was observed in the difference of estradiol level in the follicular phase and ovulation peak (*p* = 0.020 and *p* = 0.010) as well as for progesterone in the ovulation peak and the luteal phase (*p* = 0.038 and *p* = 0.032) between the study and control groups.
5. The analysed biochemical parameters related to blood coagulation, such as: D-dimer level, fibrinogen and prothrombin time, were found to be abnormal in the study group in 19.2, 19.2 and 86.5 % of cases, respectively, whereas in the control group in 18.95, 26.4 and 94.3 % of cases, respectively.
